# Monitoring of the Zeta Potential of Human Cells upon Reduction in Their Viability and Interaction with Polymers

**Published:** 2012

**Authors:** O.V. Bondar, D.V. Saifullina, I.I. Shakhmaeva, I.I. Mavlyutova, T.I. Abdullin

**Affiliations:** Kazan (Volga Region) Federal University

**Keywords:** dynamic light scattering, zeta potential, HeLa cells, MCF-7, mononuclear leukocytes, erythrocytes, apoptosis, phosphatidylserine, membranotropic polymers

## Abstract

The dynamic light scattering (DLS) technique was applied in order to assess the zeta potential of the plasma membrane of human cells. At pH 7.4, the cell zeta potential for different types of cells showed variations over a wide range and was equal to –19.4 ± 0.8 mV for HeLa cells and –31.8 ± 1.1 mV for erythrocytes. The difference could presumably be attributed to the differences in the biochemical composition of the cell plasma membrane. As a result of the heating of HeLa cells, the zeta potential shifted towards more negative voltages by 4.2 mV. An increase in the zeta potential correlated with an increase in the content of phosphatidylserine on the cell surface, which is considered to be an early marker of apoptosis. The DLS technique was also used to study the interactions between the cells and membranotropic polymers, such as polycations and nonionogenic Pluronic L121.

## INTRODUCTION

Determination of the morphological and biochemical features of human cells is necessary in order to perform an unbiased assessment of the functioning of various organs and systems of the organism [1], design new pharmaceuticals [2], and conduct fundamental research.

The parameters of living cells under normal and pathological conditions were studied using direct spectroscopy methods; i.e., the Raman, dielectric, and NMR spectroscopy techniques [3]. The cell-surface charge is the key biophysical parameter that depends on the composition of the cytoplasmic membrane and the physiological condition of cells. The cell-surface charge is assessed by measuring their electrokinetic potential (zeta potential), which characterizes the electrical double-layer potential on the cell surface. Micro-electrophoresis and capillary electrophoresis have been conventionally used to record the zeta potential of animal cells [4]; however, these techniques are labour-consuming and yield poorly reproducible results. The electrophoretic light scattering technique based on dynamic light scattering (DLS), in which the shift in the frequency or in the oscillation phase of the laser beam depends on the mobility of particles/cells in an alternating electric field [5], is a promising alternative to these methods.

The DLS technique was previously applied primarily in order to study microorganism cells [5]. We used a Zetasizer Nano ZS analyzer (Malvern Instruments) to analyze the zeta potential of animal cells.

This work was aimed at assessing the analytical capabilities of the DLS technique for determining the zeta potential of normal human cells, as well as that of human cells upon apoptosis induction, and following treatment with membranotropic polymers.

The zeta potential of human blood cells (mononuclear cells, erythrocytes) and cell lines (HeLa, MCF-7) were compared; the influence of heat-induced apoptosis and adsorption of polycations and amphiphilic nonionogenic Pluronic L121 on the zeta potential of HeLa cells was assessed.

## EXPERIMENTAL

Cell culture reagents were purchased from PanEco (Russia). Adenocarcinoma cells of uterine cervix HeLa and breast adenocarcinoma cells MCF-7 were cultured in a DMEM medium supplemented with 10% fetal bovine serum, 2 mM *L* -glutamine, 100 µg/ml streptomycin, and 100 U/ml penicillin. The cells were grown in polystyrene vials until cell monolayers were obtained. The cells were subsequently suspended using a 0.05% trypsin solution in 0.53 mM EDTA. The concentration of the suspended cells in a buffered phosphate saline (PBS) (1.7 mM KH _2_ PO _4_ , 5.2 mM Na _2_ HPO _4_ , 150 mM NaCl) was determined on a haemocytometer. Cell death was induced via heat shock (the cell suspension was heated for 30 min at 45°С).

**Fig. 1:**
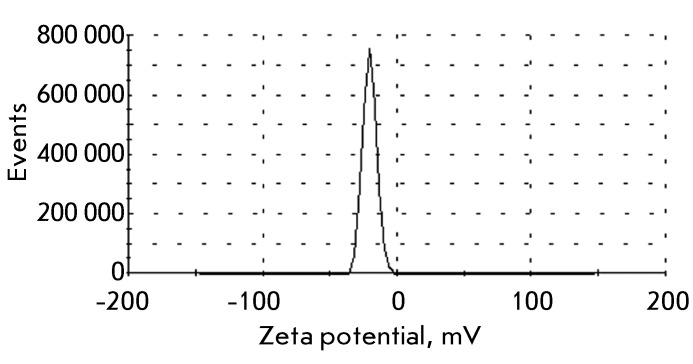
Distribution of the zeta potential in HeLa cells (0.5 × 10 ^6^ cells/ml) recorded using the dynamic light scattering technique.

**Table:** Zeta potential (ζ) of human cells and phosphatidylcholine liposomes, pH 7.4

Cells	ζ, mV*
HeLa	–19.4 ± 0.8
MCF-7	–20.9 ± 0.4
Mononuclear cells	–21.9 ± 0.2
Erythrocytes	–31.8 ± 1.1
Lyposomes (phosphatidylcholine)	–62.3 ± 1.5

*Zeta potential of cells was detected in independent triplicates. Right column shows the mean values ± standard deviation.

Erythrocytes and mononuclear cells were isolated from the peripheral blood of conventionally healthy donors; 0.27% EDTA was used as an anticoagulant agent. Blood was decanted in a vial for 60 min in order to allow the erythrocytes to precipitate. The leukocyte-containing plasma was subjected to Ficoll-Paque density gradient separation (1.077 g/ml) at 400 *g * for 40 min. The layer containing mononuclear cells was collected, washed by centrifugation, and subsequently suspended in PBS.

The zeta potential of the intact cells and those subjected to thermal shock or treatment with polymers was recorded in a suspension (0.5 × 10 ^6^ cells/ml) using the electrophoretic light scattering technique on a Zetasizer Nano ZS analyzer (Malvern Instruments, Great Britain). The measurements were performed in a U-shaped cell with gold-plated electrodes at 25°C and pH 7.4 in a phosphate buffered solution containing no chlorine ions. The results were processed using the Dispersion Technology Software 6.2 (Malvern Instruments).

Various concentrations (10, 20, 40, 50, 80 µg/ml) of polyethyleneimine (60 kDa), poly( *L* -lysine) (~20 kDa) or ethylene oxide–propylene oxide block copolymer, Pluronic L121 (Sigma-Aldrich, USA) were added to the cell suspension (0.5 × 10 ^6^ cells/ml) with the purpose of studying the interaction between the polymers and the cells. The mixture was incubated for 10 min; the cell zeta potential was subsequently determined.

In order to carry out flow cytometry, the cells were treated with a binding buffer containing FITC Annexin V and propidium iodide according to the manufacturer’s protocol (BD Biosciences, USA). The analysis was performed on a BD FACSCalibur flow cytometry apparatus (BD Biosciences); the event count was > 20,000.

## RESULTS AND DISCUSSION

The suspensions of human blood cells, as well as HeLa and MCF-7 cell lines, were used in this study. It was ascertained for HeLa cells that the zeta potential distribution curve has a maximum at –19.4 mV at pH 7.4 ( *Fig. 1* ). The zeta potential being recorded characterizes the electrical double layer potential on the cell surface [5]; its value should be dependent on the biochemical composition of plasma, provided that the solvent composition is constant.

The zeta potential values of the other cell types under study (MCF-7, mononuclear cells) were similar to that of HeLa cells, whereas the zeta potential of erythrocytes was equal to –31.9 mV ( *Table* ), which can be explained by the presence of sialic acid residues on the erythrocyte surface [6]. The negative values of the zeta potential of cell membranes at physiological pH values are presumed to be caused by the presence of nonionogenic groups within phospholipids, proteins, and their polysaccaride conjugates. The zeta potential value of phosphatidylcholine liposomes (phosphatidylcholine is the predominant lipid in animal cell membranes) is provided in the *Table * for comparative purposes. Under identical conditions, it is approximately equal to –62 mV. This fact indicates that lipids significantly contribute to the total negative charge of the cell membrane.

The changes in the zeta potential of HeLa cells subjected to heat shock were then subjected to analysis. The cell viability was assessed on a flow cytometry apparatus using a mixture of dyes, FITC Annexin V (possessing affinity to phosphatidylserine) and propidium iodide (PI), which stains necrotic cells. According to the data of flow cytometry, cell incubation at 45°С for 30 min results in the emergence of 68% of FITC-positive cells and 62% of PI-positive cells, attesting to the fact that apoptosis and cell necrosis were induced.

**Fig. 2:**
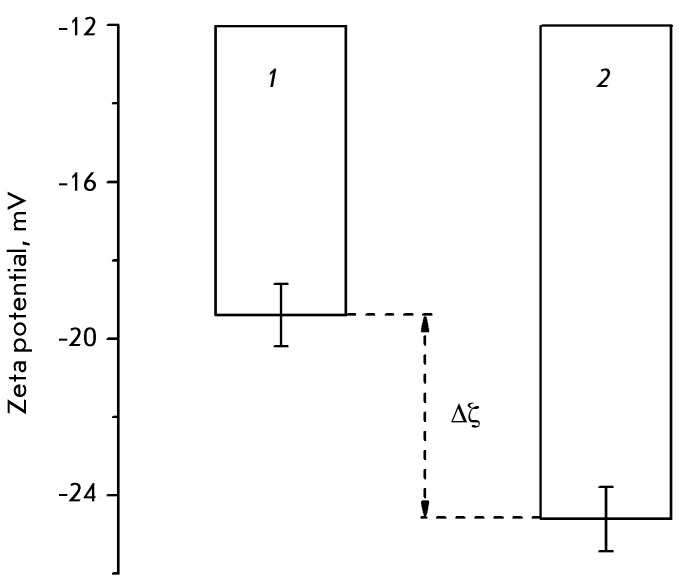
The zeta potential of HeLa cells before and after cell heating (45°С, 30 min): 1 – intact cells, 2 – heated cells, Δζ – shift of the zeta potential of the cells after the treatment.

**Fig. 3:**
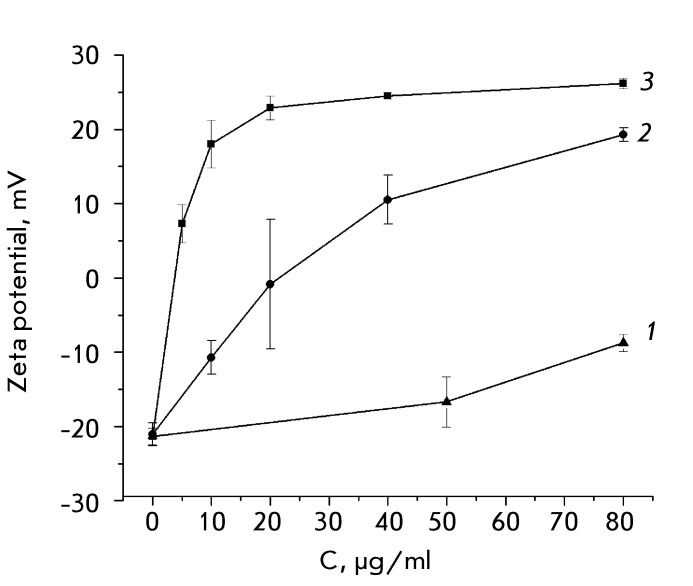
Alteration of the zeta potential in HeLa cells treated with membranotropic polymers: 1 – Pluronic L121, 2 – poly(L-lysine), 3 – polyethyleneimine.

According to the results obtained using the DLS technique, the average zeta potential of the heated cells shifted towards negative values by almost 4.2 mV, compared to the intact cells ( *Fig. 2* ). It is presumably a result of the redistribution of phosphatidylserine bearing a negatively charged carboxyl group from the inner to the outer lipid layer of the plasmalemma. The emergence of phosphatidylserine in the outer lipid monolayer of the cell membrane is one of the earliest markers of apoptosis and reduction in cell viability [6].

The results obtained attest to the fact that the DLS technique can be used to determine the changes in the biochemical composition of human cell membranes and, in particular, to detect phosphatidylserine in the outer lipid cell layer upon apoptosis induction. This approach is simple and does not require the use of expensive dyes.

It was of special interest to use the DLS technique to assess the effect of membranotropic polymers (used in cell technologies and for drug delivery) on the cell zeta potential. The interaction between HeLa cells and the model polycations, polyethyleneimine and poly( *L* -lysine), which have been widely used for DNA condensation and delivery to cells and for producing bioadhesive coatings for cell culturing [7], were studied. Another polymer, Pluronic L121, is a nonionogenic amphiphilic ethylene oxide–propylene oxide block copolymer. Block copolymers of this type are capable of irreversible interaction with cell membranes and of changing the activity of membrane transport agents, which is used to boost the efficacy of drug delivery to cells [8].

The addition of 20 µg/ml polylysine to the cells resulted in the neutralization of the cell zeta potential, presumably due to the electrostatic adsorption of the polycation on the surface of the negatively charged membrane ( *Fig. 3* ). At higher polylysine concentrations (above 20 µg/ml), the cell zeta potential becomes positive, reaching its maximum value in the presence of 80 µg/ml polylysine. Polyethyleneimine changes the charge sign of cells at a considerably lower concentration compared to polylysine (approximately 5 µg/ml), whereas the cell zeta potential is shifted to 26 mV at the saturating concentration of polyethyleneimine ( *Fig. 3* ). The effect of polyethyleneimine on the cell zeta potential is more pronounced due to the fact that this polycation is characterized by a higher density of the positive charge compared to polylysine.

The effect of Pluronic L121 on the cell surface charge was additionally assessed. Pluronic L121 is characterized by a low ratio between the hydrophilic and lipophilic parts of the molecule; its polypropylene oxide block exhibits affinity for the lipid bilayer [9]. Pluronic L121 forms nano-sized micelles in a buffered solution; according to our data, these micelles are characterized by a weakly negative zeta potential (approximately –6.7 mV).

It was ascertained that the treatment of HeLa cells with Pluronic L121 was accompanied by a noticeable shift in the cell surface charge into the positive region in proportion to the Pluronic concentration ( *Fig. 3* ). The changes observed can be attributed to the adsorption of nonionogenic Pluronic on the cell surface and the incorporation of its hydrophobic block into the membrane [10], which results in the alteration of the electrical double layer potential on the cell surface. The treatment of cells with Pluronic L121 within the analyzed concentration range did not lead to the complete neutralization of the cell charge, as was observed for polycations. Similar changes in the cell zeta potential in the presence of polymers were observed in the other cell types (MCF-7, mononuclear blood cells), which attests to the fact that the interaction between the polymers used and human cells is of a nonspecific character.

## CONCLUSIONS

The zeta potential values of blood cells (erythrocytes, mononuclear cells), as well as HeLa and MCF-7 cell lines, were determined via the DLS technique. It was shown that phosphatidylserine, the early marker of apoptosis and reduction in cell viability, can be detected based on the increase in the total negative charge of the cells. The introduction of polycations and amphiphilic Pluronic L121 results in the neutralization of the negative cell zeta potential in direct proportion to the polymer concentration.

The results demonstrate that the dynamic light scattering technique can be used to study the properties of animal cell membranes, the changes in their biochemical composition, and their interaction with membranotropic polymers under various conditions. The DLS technique can be used in cell biology to analyze the condition of the membranes of various cells, in order to assess the effect of pharmaceutical agents and membranotropic substances. 

## Abbreviations

DLS: dynamic light scattering
PBS: phosphate-buffered saline
